# The DNA cytosine methylome revealed two methylation motifs in the upstream regions of genes related to morphological and physiological differentiation in *Streptomyces coelicolor* A(3)2 M145

**DOI:** 10.1038/s41598-023-34075-1

**Published:** 2023-04-29

**Authors:** Annalisa Pisciotta, Alessia Maria Sampino, Alessandro Presentato, Marco Galardini, Angel Manteca, Rosa Alduina

**Affiliations:** 1 Department of Biological, Chemical and Pharmaceutical Sciences and Technologies (STEBICEF), University of Palermo, 90128 Palermo, Italy; 2 Department of Biology, University of Florence, Florence, Italy; 3 EMBL-EBI, Wellcome Genome Campus, Cambridge, UK; 4 Área de Microbiología, Departamento de Biología Funcional, IUOPA and ISPA, Facultad de Medicina, Universidad de Oviedo, 33006 Oviedo, Spain; 5 Present Address: Institute for Molecular Bacteriology, TWINCORE, Centre for Experimental and Clinical Infection Research, A Joint Venture Between the Helmholtz Centre for Infection Research and the Hannover Medical School, Hannover, Germany

**Keywords:** Applied microbiology, Microbial genetics

## Abstract

DNA methylation is an epigenetic modification detected in both prokaryotic and eukaryotic genomic DNAs. In bacteria, the importance of 5-methylcytosine (m5C) in gene expression has been less investigated than in eukaryotic systems. Through dot-blot analysis employing m5C antibodies against chromosomal DNA, we have previously demonstrated that m5C influences the differentiation of *Streptomyces coelicolor* A(3)2 M145 in solid sporulating and liquid non-sporulating complex media. Here, we mapped the methylated cytosines of the M145 strain growing in the defined Maltose Glutamate (MG) liquid medium. Sequencing of the M145 genome after bisulfite treatment (BS-sequencing) evidenced 3360 methylated cytosines and the two methylation motifs, GGCmCGG and GCCmCG, in the upstream regions of 321 genes. Besides, the role of cytosine methylation was investigated using the hypo-methylating agent 5′-aza-2′-deoxycytidine (5-aza-dC) in *S. coelicolor* cultures, demonstrating that m5C affects both growth and antibiotic biosynthesis. Finally, quantitative reverse-transcription polymerase-chain-reaction (RT-qPCR) analysis of genes containing the methylation motifs in the upstream regions showed that 5-aza-dC treatment influenced their transcriptional levels and those of the regulatory genes for two antibiotics. To the best of our knowledge, this is the first study that reports the cytosine methylome of *S. coelicolor* M145, supporting the crucial role ascribed to cytosine methylation in controlling bacterial gene expression.

## Introduction

DNA methylation is an epigenetic modification occurring in the genome of both prokaryotic and eukaryotic organisms. In bacteria, adenine or cytosine modification leads to the formation of N6-methyladenine (m6A), N4-methylcytosine (m4C), or C5-methylcytosine (m5C)^1^. In eukaryotic systems, the most prevalent type of DNA modification is m5C, which regulates gene expression and cellular development by altering the interaction of DNA with transcription factors or RNA polymerase^2^. Differently, in bacteria, m6A is considered a control element of gene expression in many processes^3^. Most bacterial DNA methyltransferases (MTases) are part of the restriction-modification (R-M) system. In particular, MTases protect the chromosomal DNA from the activity of the cognate restriction enzyme that destroys foreign DNA elements, such as those deriving from bacteriophages^4–6^. DNA methyltransferases that lack the cognate restriction endonucleases are termed “orphan” or solitary MTases. Orphan MTases are often involved in many cellular processes of various prokaryotes, such as the regulation of the cell cycle, DNA repair mechanisms, and the production of virulence factors in response to environmental changes^7–15^. To name a few examples, the Cell Cycle-Regulated DNA Methyltransferase (CcrM) controls the cell cycle in Alpha-proteobacteria^16^, and DNA-cytosine methyltransferase (Dcm) controls *Escherichia coli* gene expression during the stationary phase^10^.

With the advent of genome-wide molecular techniques, various studies tried to shed light on the function of epigenetic modifications in bacteria. Different procedures such as Single-molecule Real-Time (SMRT) sequencing, bisulfite [BS] sequencing, Rapid Identification of Methylase Specificity (RIMS) sequencing, and MspJI-Family Restriction Enzymes (MFRE) sequencing were adopted for the identification of the three DNA modifications (i.e., m6A, m5C, and m4C)^17–20^, with BS-sequencing considered as the gold standard to detect m5C modifications^17^.

*Streptomycetes* are Gram-positive soil bacteria with CG-rich genomes (70%). They are industrially relevant since they are prolific producers of clinically relevant secondary metabolites^21^. *Streptomyces coelicolor* A(3)2 strain M145 is the best-known species of the *Streptomyces* genus at both molecular and genetic levels^22–24^, and it has long been considered the model streptomycete for studying morphological and physiological differentiation (antibiotic production). *S. coelicolor* M145 A(3)2 produces three well-characterized antibiotics—the blue pigment actinorhodin (Act), the red one undecylprodigiosin (Red), and the calcium-dependent lipopeptide antibiotic (CDA). Besides, this strain possesses open reading frames (ORFs) encoding for up to 30 additional secondary metabolites^25^. *S. coelicolor* M145 exhibits a complex cell cycle that includes sporulation and programmed cell death^24,25^. Its life cycle is regulated at different levels by extracellular signals, quorum sensing-related factors, multiple master regulators^26^, post-translational modifications of global regulators^27^, and biochemical pathways, such as bald, white, and sky^28,29^. Also, our previous work demonstrated the correlation between m5C and the morphological and physiological differentiation in *Streptomyces coelicolor* M145 growing in solid sporulating and liquid non-sporulating complex media^30^, emphasizing the complexity level of regulatory networks.

Thus, in the present study, we carried out dot blot assays using m5C antibodies against chromosomal DNA to explore whether the *S. coelicolor* M145 genome undergoes differential DNA cytosine methylation during bacterial growth in a defined medium containing maltose and glutamate (MG medium). Also, we analyzed whether the treatment with the 5′-aza-2′-deoxycytidine (5-aza-dC) hypomethylating agent could affect growth phases and antibiotic production.

Our results show that *S. coelicolor* can control DNA cytosine methylation during its growth in the MG medium, whereas hypomethylation events induced by 5-aza-dC impair growth and antibiotic biosynthesis. Moreover, the mapping of m5C in the *S. coelicolor* M145 genome by BS-sequencing unveils two motifs (i.e., GGCmCGG and GCCmCG) in the upstream region of 321 genes. Finally, quantitative RT-PCR (RT-qPCR) of selected genes demonstrates a direct link between m5C and gene expression. To the best of our knowledge, this is the first study that reports on the cytosine methylome of *S. coelicolor* M145, deepening the current knowledge and biological significance of the methylation phenomenon triggered by this streptomycete strain.

## Results

### Growth profile, cytosine methylation level, and the effect of cytosine hypomethylation and de-methylation on growth and physiological differentiation in *S. coelicolor* M145

**Figure 1:**
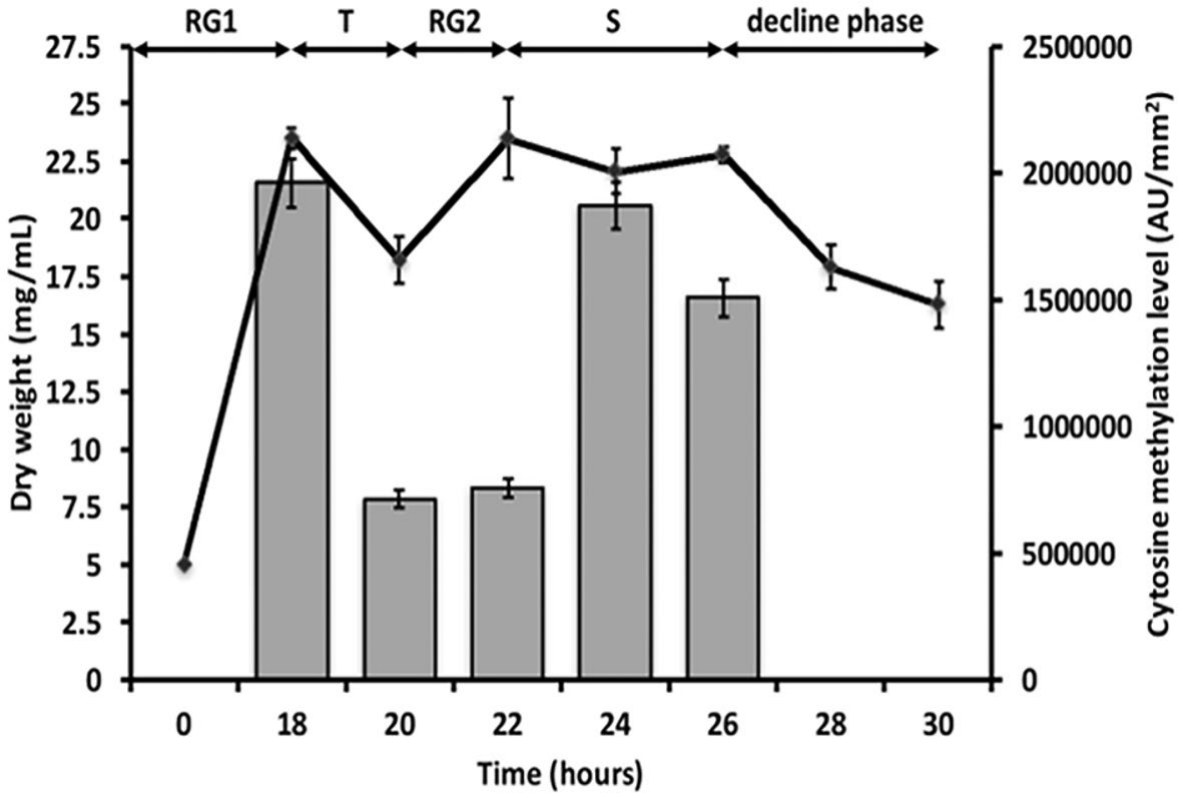
Growth curve of *S. coelicolor* M145 in the MG medium (continuous curve) and DNA cytosine methylation level (grey bars). The growth was evaluated through evaluation of the dry weight and the methylation level was measured by dot blot analysis.

The growth profile of *S. coelicolor* M145 was evaluated in MG medium by using the dry weight method, specifically *S. coelicolor* M145 shows a typical biphasic growth curve (Fig. 1), characterized by two rapid growth phases (RG1 and RG2), a transition (T), and a decline phase (D)^31^. The transition phase (T) corresponds to the transient arrest of the cell growth, allowing macromolecular biosynthesis, the turnover of ribosomal proteins, and the onset of secondary metabolite production (e.g., antibiotics)^32^. Genomic DNA was extracted at different time points (i.e., 18, 20, 22, 24, and 26 h) of *S. coelicolor* M145 growth and analyzed by dot blot assay using the antibody against m5C. This analysis showed that the levels of m5C were higher at 18, 24, and 26 h than at 20 and 22 h (Fig. 1).

**Figure 2:**
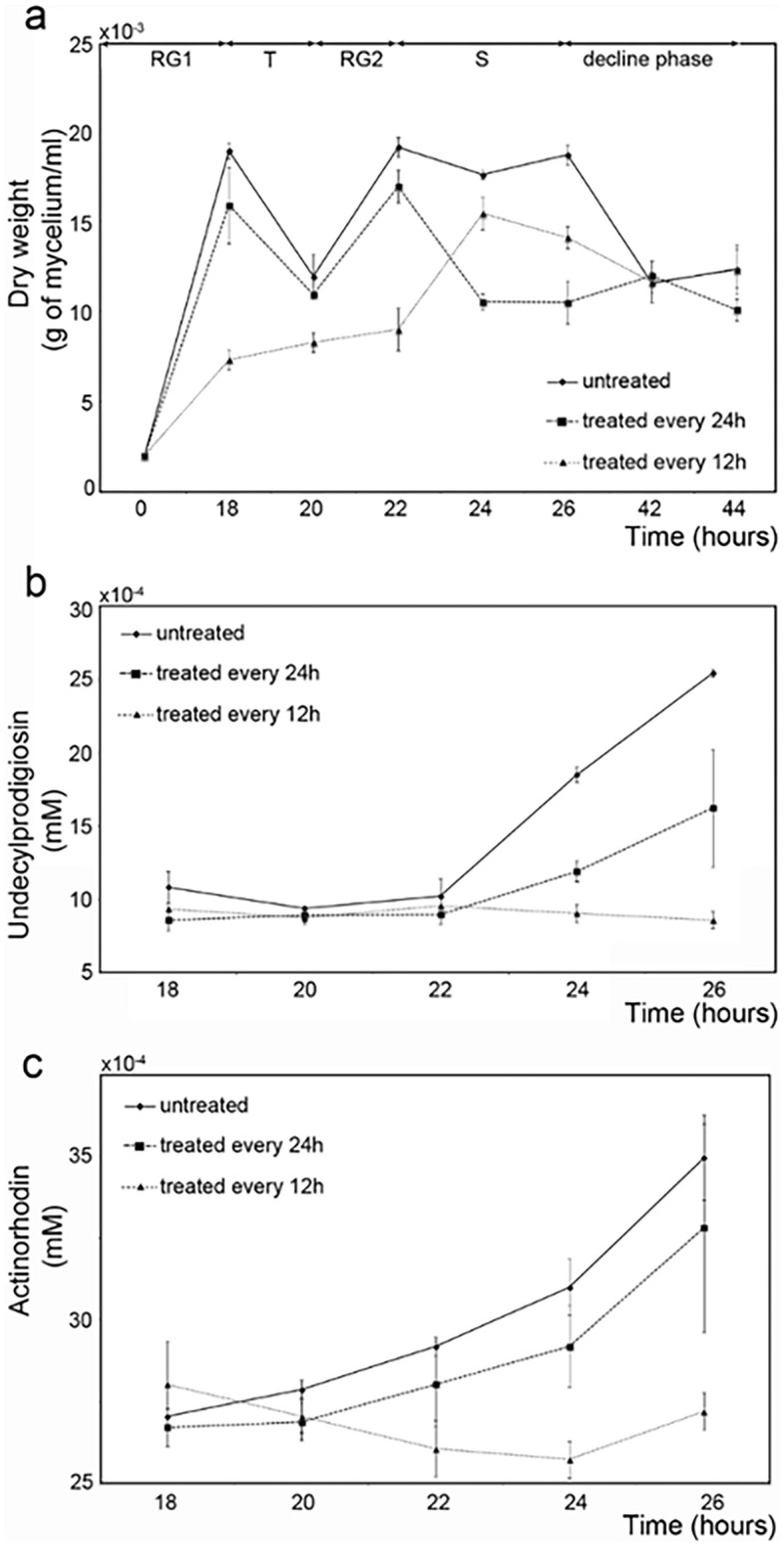
Growth curves (**a**) and quantitative analysis of undecylprodigiosin (**b**) and actinorhodin (**c**) of *S. coelicolor* M145 cultures untreated (indicated by circles) and treated with 5-aza-dC every either 12 (indicated by triangles) or 24 h (indicated by squares).

The effect of cytosine methylation on morphological and physiological differentiation of the *S. coelicolor* M145 strain was evaluated by treating the cells with the hypomethylating agent 5′-aza-2′-deoxycytidine (5-aza-dC). Since the estimated half-life of 5-aza-dC is 20–24 h^33^, the compound was added to *S. coelicolor* M145 cultures every either 12 or 24 h, starting from the time of inoculum. The 24 h-treatment resulted in 72% of demethylation, while the 12 h-treatment allowed to achieve a 99.5% of demethylation. As a consequence, the 12 h-treatment strongly affected cell growth, even though bacterial cells were still viable (Fig. 2a). Indeed, *S. coelicolor* M145 cells featured a growth curve with an evident impairment of the RG1 phase, followed by an arrest of the bacterial growth before the cells enter the RG2 phase, which was delayed in time as compared to the untreated cells (Fig. 2a). On the other hand, the *S. coelicolor* M145 strain treated every 24 h with 5-aza-dC showed a growth profile that resembled that of the untreated cells up to 22 h incubation: after a 2 h-decline phase, cells enter a stationary phase until the end of the considered timeframe (Fig. 2a).

The production of undecylprodigiosin and actinorhodin by the 12 h-treated cells was strongly impaired at 24 and 26 h of growth. Inhibition of the antibiotic biosynthesis was less pronounced in the 24 h-treated cultures, being only undecylprodigiosin production significantly influenced from 24 h of growth onwards (Fig. 2b). As for actinorhodin, any statistically valid fluctuation in antibiotic production was not observed as compared to the untreated cultures (Fig. 2c).

Overall, 5-aza-dC needs to be added to the cultures every 12 h to show a significant decrease in the number of methylated cytosines and, as a consequence, strong effects on *S. coelicolor* growth and antibiotic production.

### Mapping of m5C by BS-sequencing

**Figure 3:**
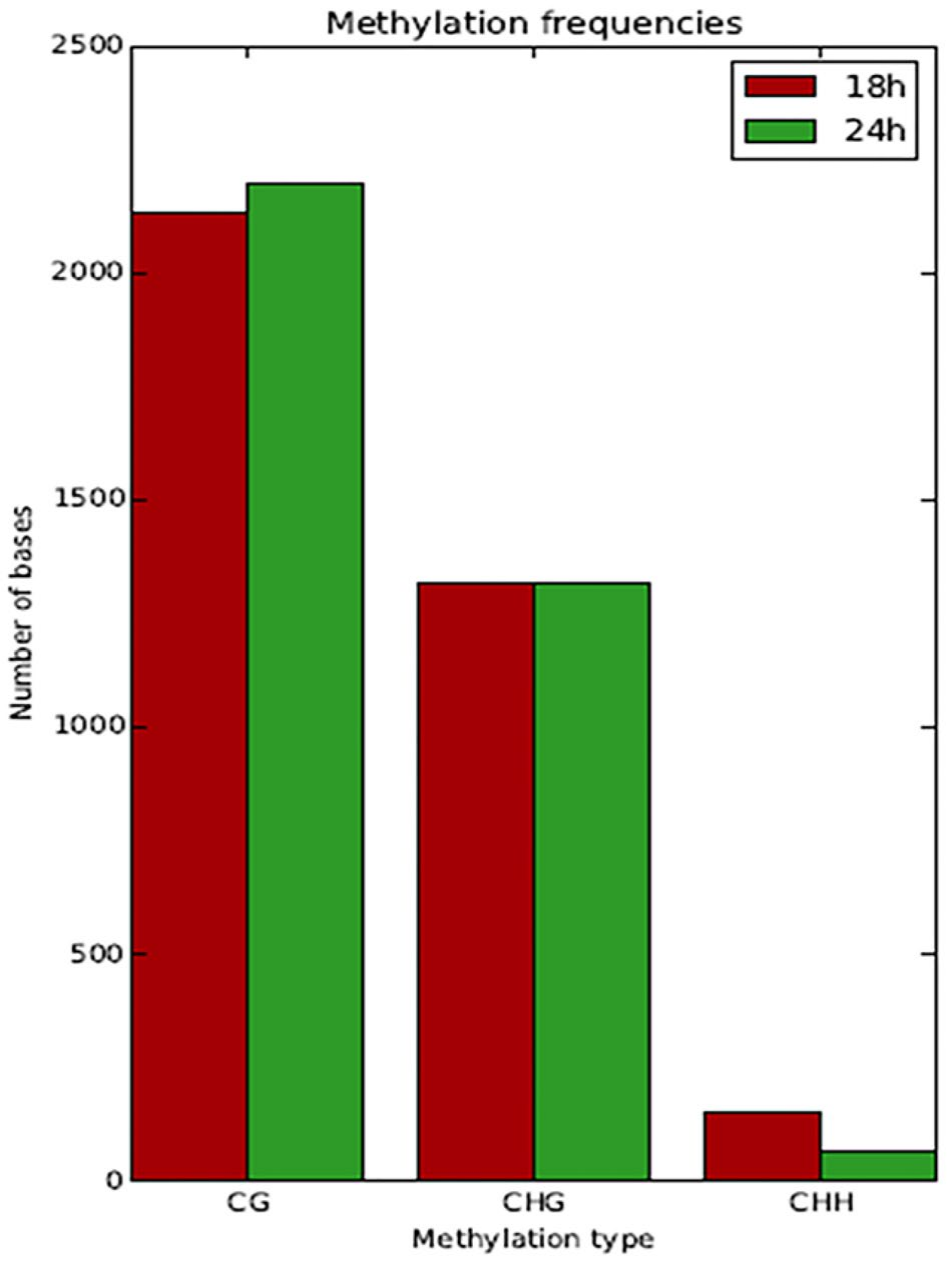
Number of methylated bases based on the CmG, CmHG, and CmHH motifs found in *S. coelicolor* M145 genome after 18 and 24 h of growth in MG. H stands for A, T, or C.

Since higher m5C levels were detected at 18 and 24 h of the bacterial growth in the MG medium (Fig. 1), methylome analysis by BS-sequencing was carried out on genomic DNA extracted from cells collected at these time points. BS-sequencing demonstrated that 3360 cytosines of the genome were methylated, and the most frequent methylation motif was CmG, followed by the CmHG and CmHH (Fig. 3). The differences between DNA samples extracted at 18 and 24 h of growth were minimal, with slightly high methylation of CmHG and low CmHH for the 24 h sample.

**Figure 4:**
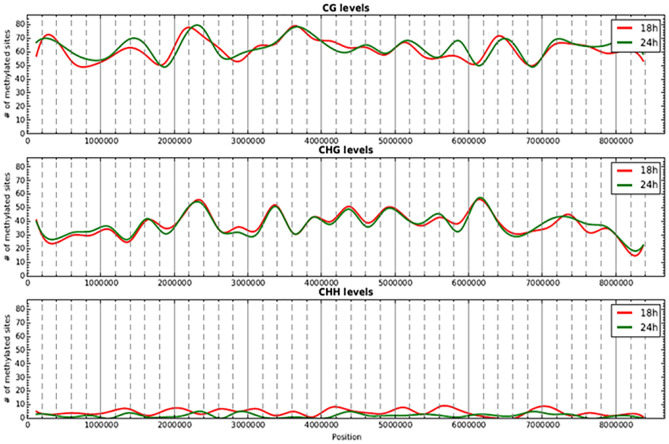
Methylation levels along the chromosome (250 kbp window) at 18 and 24 h of growth in the MG medium. Red and green lines correspond to the methylation levels at 18 h and 24 h, respectively.

The distribution of the methylated cytosines on the *S. coelicolor* M145 chromosome appeared homogeneous, with a few hot regions (Fig. 4).

**Figure 5:**
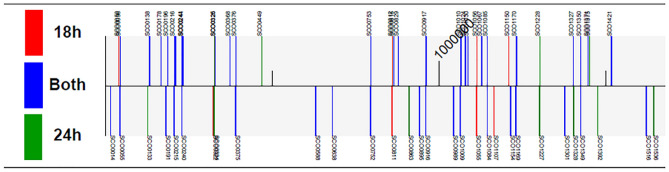
Plot of the methylated cytosines at 18 and 24 h of growth of the M145 strain in the MG medium.

In the search for genes containing the above-mentioned methylation motifs, we arbitrarily considered 500 bp upstream of the putative translation start site of each gene to 100 bp downstream, retrieving a total of 321 genes (Table S1) sharing two consensus sequences, GGCmCGG and GCCmCG. The position of the methylated bases on the chromosome of *S. coelicolor*, as well as that of genes whose upstream regions contained at least one methylated base, appeared to be homogeneously distributed (Fig. 5), even though some DNA regions were methylated-cytosines free.

### DNA methylation and gene expression

Among the 321 genes, we found genes coding sequences that code for proteins involved in either primary metabolism (i.e., oxidoreductases, beta-mannosidases, and proteinases, to name a few) or, in secondary (i.e., dihydrodipicolinate synthase [DapA], serine hydroxymethyltransferase [Glya1], 3-methyl-2-oxobutanoate hydroxymethyltransferase [PanB], aspartate-semialdehyde dehydrogenase [Asd1], lycopene cyclase [CrtY], nucleotide-sugar dehydratase [CwgA]), proteins those related to DNA/RNA metabolism, putative transcriptional regulators and sigma factors, membrane proteins including ABC transporters, penicillin-binding protein and hypothetical proteins (Fig. S1).

To correlate DNA cytosine methylation and gene expression, the transcriptional analysis of ten genes containing methylated cytosines in their upstream region was performed by quantitative reverse transcription RT-qPCR analysis (Table 1). Specifically, the gene expression was evaluated after 5-aza-dC treatment carried out every 12 h since the latter affected antibiotic production and growth more than the treatment every 24 h (Fig. 2b,c).

**Table 1:** List of the genes containing methylated cytosines in their upstream region chosen for RT-qPCR analysis.

Gene	Cellular function	Time of methylation (h)
*SCO6164*	Molecular chaperone DnaK-like	24
*SCO6685*	Two-component system response regulator (RamR)	24
*SCO5820*	RNA polymerase sigma factor (HrdB)	18–24
*SCO2950*	DNA-binding protein HU (Hs1)	18–24
*SCO2571*	Leucyl-tRNA synthetase	18–24
*SCO2077*	Determinant of *Streptomyces* apical growth and hyphal branching (DivIVA)	18–24
*SCO2964*	LysR family transcriptional regulator (StgR)	18–24
*SCO3911*	Replicative DNA helicase (DnaB)	18–24
*SCO3732*	DEAD/DEAH box helicase	18–24
*SCO2716*	Secreted hydrophobic protein (ChpA)	18–24

**Figure 6:**
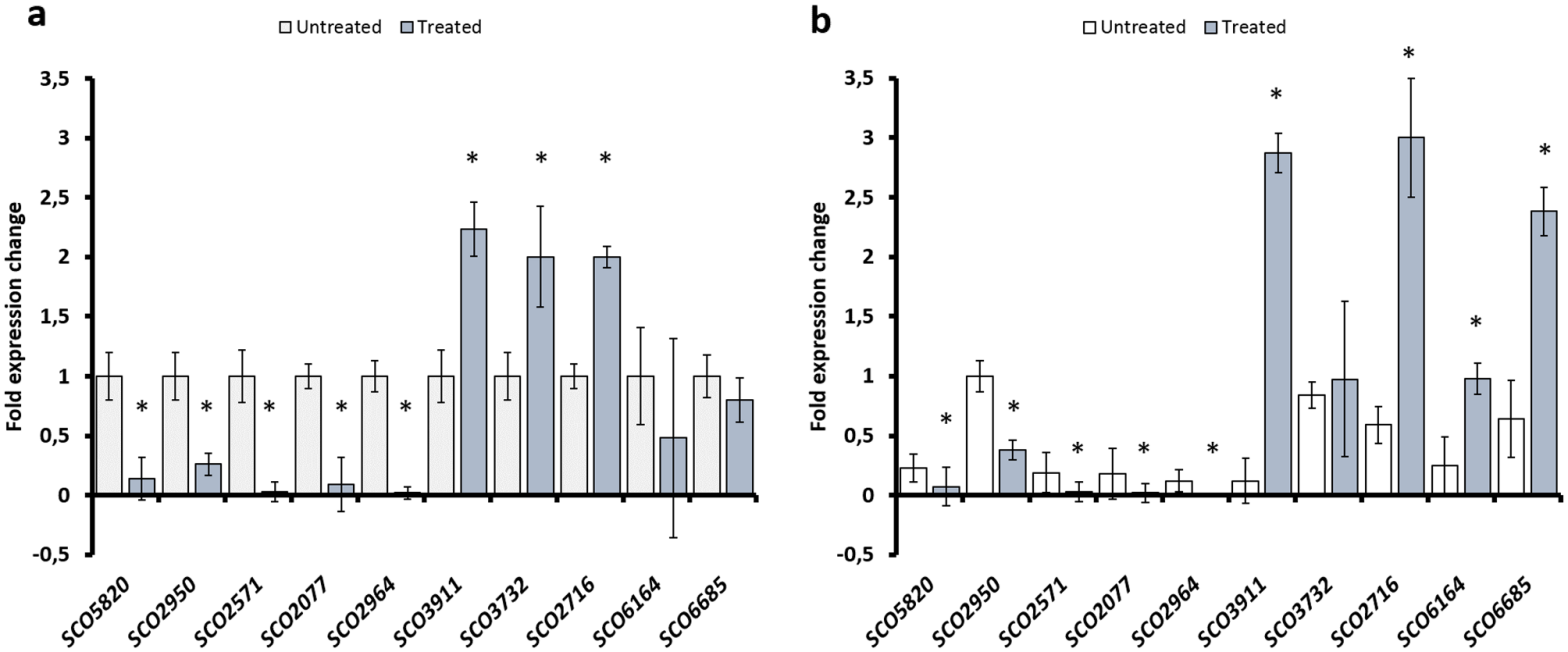
RT-qPCR analysis of *SCO5820, SCO2960, SCO2571, SCO2072, SCO2964, SCO3911, SCO3732, SCO2716, SCO6164,* and *SCO6685* genes after 18 h (**a**) and 24 h (**b**) of growth of untreated and 5-aza-dC treated cultures. mRNA levels are expressed as relative values to 16S rRNA transcripts, with the ratio values for the 18 h sample arbitrarily set to 1. The standard deviations (indicated by error bars) were calculated from three independent determinations of mRNA abundance. The asterisks indicate significant differences compared to the untreated sample (*p*-value equal to or less than 0.05).

We found a different effect of demethylation on gene transcription levels of the tested genes. Specifically, we obtained three different transcriptional responses to the 5-aza-dC treatment. Group 1 contains *SCO5820*, *SCO2950*, *SCO2571*, *SCO2077*, and *SCO2964* genes, whose transcription was down-regulated by the 5-aza-dC treatment at 18 (Fig. 6a) and 24 h (Fig. 6b) of growth. These genes contain the methylated motif in their upstream regions at both time points of bacterial growth. Group 2 contains the *SCO3911*, *SCO3732*, and *SCO2716* genes, whose transcription was enhanced by the 5-aza-dC treatment for each incubation time considered, although for *SCO3732* the difference at 24 h was not statistically relevant (Fig. 6a,b). The third group contains the *SCO6164* and *SCO6685* genes, for which an increased transcription level was observed only at 24 h of growth (Fig. 6b), being their upstream region only methylated at this growth stage of the M145 strain.

### Effect of 5-aza-dC on the positive regulators of undecylprodigiosin and actinorhodin

**Figure 7:**
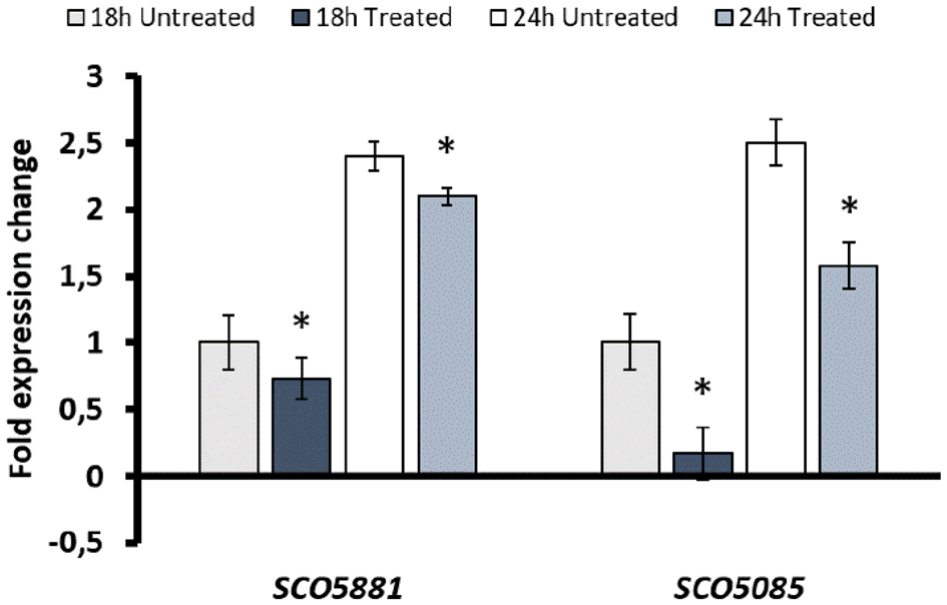
RT-qPCR analysis of *SCO5881* and *SCO5085* at 18 and 24 h in the untreated and treated cultures The asterisks indicate significant differences compared to the untreated sample (p-value equal to or less than 0.05).

Since 5-aza-dC treatment determined an impairment of undecylprodigiosin and actinorhodin biosynthesis, gene expression analysis of positive regulatory ones [i.e., *SCO5881* (*redZ*) and *SCO5085* (*actII-orf4*)] was investigated^34^. Also, it is worth mentioning that the upstream region of these genes did not feature any methylated cytosines. Specifically, RT-qPCR was performed on transcripts isolated from untreated and 12 h-treated M145 cultures at 18 and 24 h of bacterial growth. In the untreated culture, RT-qPCR analyses showed that both genes were up-regulated at 24 h compared to the 18 h incubation time. The hypomethylating agent 5-aza-dC led to a slight down-expression of *SCO5881* transcript (~ 0.27 at 18 h and ~ 0, threefold at 24 h); this phenomenon was emphasized in the case of *SCO5085* for each considered time point (~ 0.83 and ~ 0.92 fold respectively at 18 and 24 h) (Fig. 7). These data suggested an indirect effect of m5C DNA methylation on the biosynthesis of secondary metabolites by altering transcriptional levels of the positive regulators.

## Discussion

In our precedent study, we established a previously unknown link between DNA cytosine methylation (m5C) and physio-morphological differentiation in *S. coelicolor* M145^30^, suggesting that DNA cytosine methylation could be a still unexplored regulatory mechanism in this model strain. Indeed, by decreasing the cytosine methylation level using a hypomethylation agent or by deleting the *SCO1731* gene coding for a methyltransferase, a series of striking effects were noted, such as delay in spore germination, mycelium differentiation, and sporulation, alongside the delayed undecylprodigiosin production and impairment of actinorhodin synthesis. The dot blot assay showed that cytosine methylation is controlled during *S. coelicolor* M145 growth in the defined MG medium (Fig. 1), which is in line with the results we got in other growth media, the liquid R5A and solid GYM^29^. In the latter, a higher level of m5C was detected during the MI stage. Moreover, bacterial cells experiencing hypomethylating effects of 5-aza-dC, which alters the regular DNA cytosine methylation pattern, resulted in impaired growth and antibiotic production (Fig. 2), as earlier observed for other media^30^. Here, the m5C mapping in the *S. coelicolor* M145 genome revealed two methylation consensus sequences in the upstream region of 321 genes. As far as we know, epigenetic modifications—cytosine and adenine methylation—have been described and considered responsible for controlling secondary metabolism only in *Streptomyces roseosporus* L30, where a consensus sequence for m6A and a priority of the GCGG motif for m4C were identified^35^. Furthermore, transcriptomic data on the mutant strain of *S. roseosporus* for m4C methyltransferase (*SroLm3*) revealed an upregulation of genes encoding an ABC transporter and ribosomal proteins. Recently, a potential connection between DNA methyl modification and the synthesis of erythromycin A in *Saccharopolyspora erythraea* was discussed^36^. We cannot rule out that other modifications to *S. coelicolor* genomic DNA can occur, given the specificity of BS-sequencing for detecting m5C^17^.

Amongst the genes containing methylated cytosines in the upstream region, bioinformatic analysis identified genes that code for proteins involved in primary and secondary metabolism. According to the data observed, these genes are correlated with growth and differentiation in *S. coelicolor* M145, underlining the possible role of DNA cytosine methylation on gene expression regulation. The methylated base could interfere with the binding of transcription factors at the regulatory region of a gene, activating or inhibiting the gene transcription^4^. Indeed, RT-qPCR analysis showed how the transcription of genes containing methylation motifs in the upstream region and involved in amino acid metabolism, DNA/RNA metabolism, and gene expression regulation was altered differently upon treatment of *S. coelicolor* cells with 5-aza-dC (Fig. 6). The hypomethylating agent downregulated the gene expression of five genes involved in *Streptomyces'* primary and secondary metabolism. Specifically, it impaired the gene expression of *SCO5820*, encoding for the principal sigma factor (HrdB), agreeing with the reduced growth of the treated culture (Fig. 2a)*.* Coherently, previous studies showed that HrdB controls the transcription of 56% of genes involved in the energy metabolism of *Streptomyces*^37^. In addition, the hypomethylation due to the 5-aza-dC downregulated the transcriptional level of *SCO2964*, encoding for a LysR-like regulator. In our experimental setup, it seems that the *SCO2964* downregulation leads to a reduction of antibiotic production in *S. coelicolor*. This result is in contrast with the fact that regulators belonging to the LysR protein family act as repressors of actinorhodin production in both *S. coelicolor* and *S. lividans*^38–40^. Also, the regulatory activity of *SCO2964* might be modulated by post-translational modifications, similar to the acetylation of the global regulator GlnR, whose modification affects the activity of this transcriptional regulator^29^. However, other studies are necessary to understand the role of this LysR-like regulator in *Streptomyces* and how post-translational modifications can influence the activity of such regulatory proteins. Contrarily, 5-aza-dC treatment upregulated the transcription level of *SCO3911*, and *SCO3732*, which are involved in DNA metabolism, thus allowing us to assume that an alteration of the stability of replication machinery may be toxic to the cell leading to death events in the treated culture than in the untreated one. In addition, the hypomethylation upregulates the expression of *SCO2716,* encoding for a ChpA protein involved in aerial hyphae formation, *SCO6164*, encoding a hypothetical protein, and *SCO6685,* encoding a two-component system response regulator. The upregulation of the ChpA protein (*SCO2716*) was reported to reduce the actinorhodin and undecylprodigiosin production in *S. coelicolor*^41^. Thus, this result will confirm the role of cytosine methylation in controlling antibiotic biosynthesis and regulating the expression of specific genes.

In summary, this is the first comprehensive report on genome-wide cytosine methylation in *S. coelicolor* M145. The present methylome data show that DNA cytosine methylation occurs in the upstream region of genes involved in primary and secondary metabolism, transport, two-component system, and signal transduction. This data indicates that the m5C DNA methylation can directly regulate the biosynthesis of secondary metabolites through modulation of gene expression, but the molecular mechanism needs to be explored in the future.

## Materials and methods

### Strains and media

*Streptomyces coelicolor* A(3)2 strain M145 was cultured by inoculating 100 μl of a spore suspension (1 × 10^8^ viable spores/ml) in 250 ml flasks containing 25 ml JM medium (sucrose 100 g/L, tryptone soya broth 30 g/L, yeast extract 10 g/L, and MgCI_2_.6H_2_0 10 g/L). The culture was grown at 200 rpm and 30 °C in an orbital shaker for 48 h. Afterward, mycelium was harvested by centrifugation at 3,000 xg for 15 min, washed twice in water, and resuspended in 50 ml of water. From this suspension, 5 ml was inoculated into 200 ml of MG medium (maltose and glutamate)^31^ and cultivated as earlier described. The mycelium of *S. coelicolor* M145 was collected at different time points (from 0 to 30 h), being the bacterial growth profile estimated by the dry weight method. Three independent cultures were prepared, and genomic DNA was extracted by salting out procedures^42^ at different time points of growth. DNA quantification was performed via NanoDrop ND1000 Spectrophotometer.

### 5-aza-dC treatment

Experiments for the set-up of the cytosine DNA hypomethylation treatment were carried out as previously described^30^. 5-aza-dC is reported to have a half-life of 20 h-24 h under physiological temperature and neutral pH conditions^31^. Thus, 5-aza-dC treatment was performed by adding 5 µM of this compound every 24 h to achieve hypomethylation, while a subsequent addition every 12 h was necessary to get an almost total de-methylation, which was evaluated by dot blot assay^30^. Treatment was carried out in liquid cultures every 12 and 24 h, from 0 to 44 h.

### Antibiotic quantification

Undecylprodigiosin and actinorhodin were quantified spectrophotometrically as described in Pisciotta et al., 2018^30^. Reproducibility has been corroborated by at least three independent cultures at various growth stages of bacterial incubation.

### Dot Blot assay and Bisulfite sequencing

Genomic DNA was extracted by phenol extraction and purified by GenElute™ Bacterial Genomic DNA Kit (Sigma Aldrich). The concentration and quality of DNA samples were measured by NanoDrop ND1000 Spectrophotometer. Aliquots of genomic DNA were tested by dot blot assay using the protocols described by Caracappa et al.^43^. Genomic DNA was dried and sent to BGI Honk Hong CO. DNA sequencing was carried out by Illumina’s HiSeq Technology. The obtained genomic sequences were compared to the annotated *S. coelicolor* M145 genome sequence (link http://www.ncbi.nlm.nih.gov/nuccore/NC_003888.3). The data related to the methylated sequences have been deposited under the accession number BioProject ID PRJNA933392. Data were analyzed and visualized using the following python libraries: Python 2.7, iPython 1.0.0 (25), biopython 1.62 (26), Matplotlib 1.2.1 (27), NumPy (28), and SciPy.

### Real-time quantitative reverse transcription PCR (RT-qPCR)

Bacterial cells grown for 18 and 24 h in the presence/absence of 5 µM 5-azadC were harvested to isolate total RNA. The cells were broken by using 1 mg of lysozyme/ml in P buffer, and total RNA was isolated by using the RNeasy mini-kit (QIAGEN), as reported in^44^. DNase I (Roche) treatment was performed at 37 °C for 1 h, and ethanol precipitation in the presence of 0.1 vol 3 M sodium acetate allowed recovery of the DNase-treated total RNA. After a washing step with 70% ethanol and air drying, the RNA pellet was resuspended in RNAse-free water. RT-qPCR was performed by using a Superscript One-Step RT-PCR kit (Invitrogen) with about 0.1 µg of total RNA as a template, primer pairs internal to genes of interest (Table S2), and the conditions indicated by the supplier, routinely using 40 PCR cycles. For each reaction, negative control with Taq polymerase and without reverse transcriptase was included. Expression was analyzed quantitatively by PCR using the Applied Biosystems 7300 real-time PCR system (Applied Biosystems). A high-capacity cDNA archive kit (Applied Biosystems) was used, according to the manufacturer’s instructions, to retro transcribed 5 µg of total RNA, extracted after 18 and 24 h of growth from untreated and treated with 5-aza-dC cultures, in a final volume of 100 µl of water. Then, 3 µl of the cDNA was mixed with a 10 µl SYBR green PCR master mix (Applied Biosystem) and 10 pmol of each primer in a final volume of 20 µl. The PCR was performed under the following conditions: 2 min at 50 °C and 10 min at 95 °C, followed by 40 cycles of 15 s at 95 °C and m1 min at 68 °C. Finally, a dissociation reaction was performed with the following conditions: a 1-min step with a temperature gradient increase of 1 °C per step from 55 to 99 °C. This last reaction allowed the melting curve of the PCR products and, consequently, their specificity to be determined. A negative control (distilled water) was included in all real-time PCR assays, and each experiment was performed in triplicate. The 16S rDNA gene was used as an internal control to quantify the relative expression of target genes.

## Supplementary Information

Supplementary Information.

## Data Availability

The data related to the methylated sequences have been deposited under the accession number BioProject ID PRJNA933392.
