# Supplementary material for: The DNA cytosine methylome revealed two methylation motifs in the upstream regions of genes related to morphological and physiological differentiation in Streptomyces coelicolor A(3)2 M145
Source: Sci Rep. 2023 Apr 29;13:7038. doi: 10.1038/s41598-023-34075-1 (PMC10148868; doi:10.1038/s41598-023-34075-1)
Supplement: Supplementary file 1 — Supplementary Information. [file 41598_2023_34075_MOESM1_ESM.pdf]

The DNA cytosine methylome revealed two methylation motifs in the upstream regions of genes related to morphological and physiological differentiation in *Streptomyces coelicolor* A(3)2 M145

Annalisa Pisciotta[1], Alessia Maria Sampino[1], Alessandro Presentato[1], Marco Galardini[2,3,#], Angel Manteca[4], Rosa Alduina[1*]

Affiliations;

1) Department of Biological, Chemical and Pharmaceutical Sciences and Technologies (STEBICEF), University of Palermo, 90028 Palermo, Italy.

2) Department of Biology, University of Florence, Florence, Italy

3) EMBL-EBI, Wellcome Genome Campus, Cambridge, United Kingdom

4) Área de Microbiología, Departamento de Biología Funcional, IUOPA and ISPA, Facultad de Medicina, Universidad de Oviedo, 33006 Oviedo, Spain

#) Current address: Institute for Molecular Bacteriology, TWINCORE, Centre for Experimental and Clinical lnfection Research, a joint venture between the Helmholtz Centre for Infection Research and the Hannover Medical School, Hannover, Germany

Corresponding author: Rosa Alduina, email: valeria.alduina@unipa.it

Supplementary Figure S1: Function of genes containing a methylated cytosine in the upstream region.

Supplementary Table S1: List of the genes containing methylated cytosines in their upstream genes. The gene functions and the methylated sequences are reported.

Supplementary Table S2: Primers sequence

Figure S1 Function of genes containing methylated cytosine in their upstream region

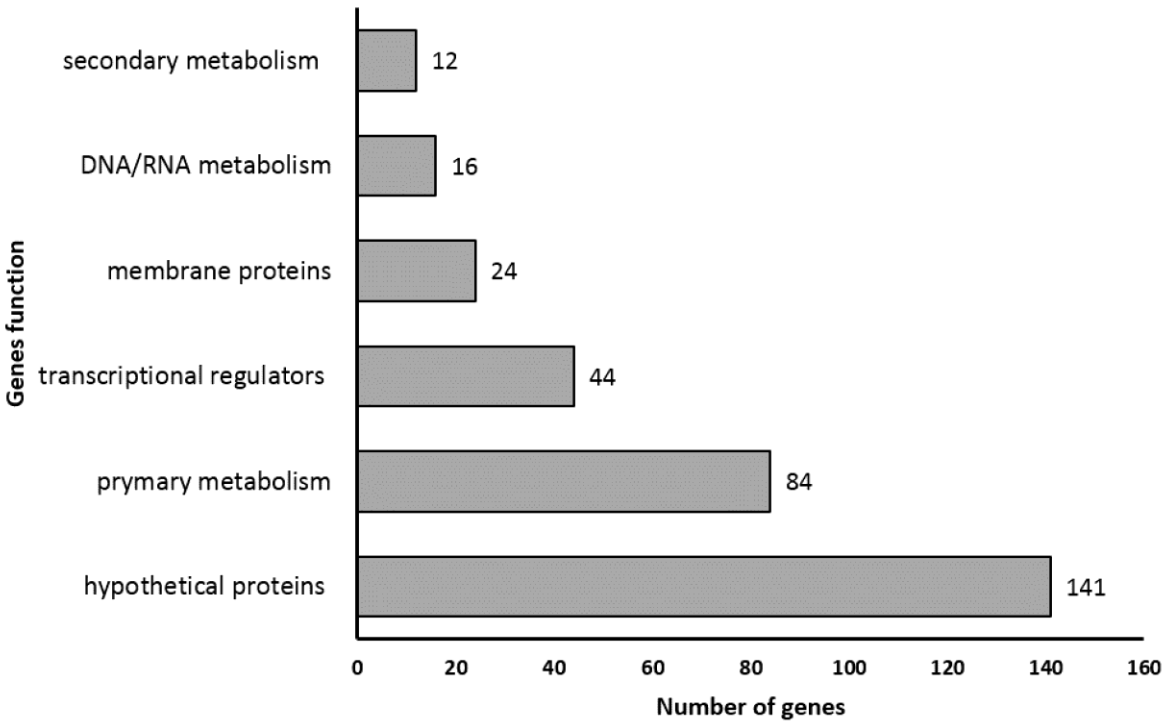

Table S1 List of the genes containing methylated cytosines in their upstream genes. The gene functions and the methylated sequences are reported.

		18h			24h			18h			24h			18h			24h		
Gene	Description	CG	CHG	CHH	CG	CHG	CHH	CG	CHG	CHH	CG	CHG	CHH	CG	CHG	CHH	CG	CHG	CHH
SCO7197	amino acid ABC transporter permease	GCCGGC	AGCCCG		GCCGGC	AGCCCG		+	-		+	-		-182	-130		-182	-130	
SCO5289	two component sensor kinase	GGCCGG			GGCCGG			-			-			-104			-104		
SCO1327	hypothetical protein	GCCGGC			GCCGGC			+			+			-183			-183		
SCO0376	transcriptional regulator		GCCCGC			GCCCGC			+			+			-22			-22	
SCO4146	ECF family RNA polymerase sigma factor	CCCGCC						+						-244					
SCO6723	oxidoreductase		AGCCCG			AGCCCG			-			-			-297			-297	
SCO2964	LysR family transcriptional regulator (stgR)	GGCCGG			GGCCGG			-			-			-196			-196		
SCO5439	hypothetical protein	GCCGGC			GCCGGC			+			+			-112			-112		
SCO6234	beta-mannosidase		GCCCGC			GCCCGC			+			+			-161			-161	
SCO2112	hypothetical protein		GCCCGC			GCCCGC			+			+			-65			-65	
SCO2113	bacterioferritin		GCCCGC			GCCCGC			+			+			-126			-126	
SCO1154	hypothetical protein	GCCGGC			GCCGGC			+			+			-29			-29		
SCO2571	leucyl-tRNA synthetase		AGCCCG			AGCCCG			-			-			-227			-227	
SCO4756	hypothetical protein		GCCCGG			GCCCGG			+			+			-28			-28	
SCO6909	hypothetical protein		AGCCCG			AGCCCG			-			-			-177			-177	
SCO4755	transcriptional regulator		GCCCGA			GCCCGA			+			+			-60			-60	
SCO5027	hypothetical protein		AGCCCG			AGCCCG			-			-			-105			-105	
SCO1010	integral membrane transport protein	GCCGGC<br>GCCGGC			GCCGGC<br>GCCGGC			+ +			+ +			-302<br>-264			-302<br>-264		
SCO4293	threonine synthase				GTCGTG						+						-39		
SCO6164	hypothetical protein					ACCAGA						+						-235	
SCO5820	RNA polymerase sigma factor	GCCGGC<br>GGCCGG			GGCCGG<br>GCCGGC			+ -			- +			-369<br>-368			-368<br>-369		
SCO3540	proteinase		AGCCCG			AGCCCG			-			-			-102			-102	
SCO3109	transcriptional-repair coupling factor	GCCGGC			GCCGGC			+			+			-132			-132		
SCO7637	endoglucanase	GGCCGG			GGCCGG			-			-			-322			-322		
SCO0050	hypothetical protein			GCCACG						+						-53			
SCO3732	DEAD/DEAH box helicase	GAACGC			GAACGC			-			-			-141			-141		
SCO5610	hypothetical protein		AGCCCG			AGCCCG			-			-			-3			-3	

SCO1328	hypothetical protein					CACTGG						+						-229	
SCO5614	transcriptional regulator		GCCCGG			GCCCGG			+			+			-2			-2	
SCO0133	hypothetical protein						CCCCAT						-						-153
SCO4662	elongation factor Tu		AGCCCG			AGCCCG			-			-			-68			-68	
SCO5619	hypothetical protein	GGCCGG			GGCCGG			-			-			-307			-307		
SCO1955	iron sulfur binding protein	GTCGGG						+						-16					
SCO7628	hypothetical protein	GCCGGC			GCCGGC			+			+			-99			-99		
SCO5767	hypothetical protein				GGCCGG						-						-41		
SCO2965	transporter	GGCCGG			GGCCGG			-			-			-82			-82		
SCO2914	amino acid permease	GCCGGC			GCCGGC			+			+			-114			-114		
SCO7608	hypothetical protein	TCCGCC						+						-10					
SCO6347	beta-galactosidase	GCCGGC			GCCGGC			+			+			-46			-46		
SCO3474	sugar kinase		AGCCCG			AGCCCG			-			-			-3			-3	
SCO4441	DNA-binding protein		GCCCGG			GCCCGG			+			+			-136			-136	
SCO7445	hypothetical protein	GGCCGG			GGCCGG			-			-			-174			-174		
SCO2046	integral membrane efflux protein	GCCGGC			GCCGGC			+			+			-91			-91		
SCO0240	oxidoreductase		AGCCCG			AGCCCG			-			-			-44			-44	
SCO0241	transcriptional regulator		AGCCCG			AGCCCG			-			-			-301			-301	
SCO7295	LuxR family transcriptional regulator		AGCCCG			AGCCCG			-			-			-24			-24	
SCO0244	hypothetical protein		GCCCGG			GCCCGG			+			+			-46			-46	
SCO2971	hypothetical protein		CCCAGA						+						-108				
SCO3801	aminopeptidase 2		GCCCGT			GCCCGT			+			+			-76			-76	
SCO1985	hypothetical protein		AGCCCG			AGCCCG			-			-			-115			-115	
SCO6380	hypothetical protein	GCCGGC			GCCGGC			+			+			-53			-53		
SCO1392	hypothetical protein				GTCGGG						+						-66		
SCO3577	ion-transporting ATPase	GCCGCC						+						-88					
SCO3576	hypothetical protein	GCCGCC						+						-64					
SCO2572	hypothetical protein		AGCCCG			AGCCCG			-			-			-309			-309	
SCO1067	integral membrane transport protein		GCCCGA		CCACGG	GCCCGA			+		-	+			-15		-95	-15	
SCO7607	hydrolase	TCCGCC						+						-56					
SCO3760	hypothetical protein		GCCCGG			GCCCGG			+			+			-89			-89	
SCO3888	hypothetical protein		AGCCCG			AGCCCG			-			-			-109			-109	
SCO4280	reductase		GCCCGG			GCCCGG			+			+			-149			-149	
SCO4281	hypothetical protein		GCCCGG			GCCCGG			+			+			-186			-186	
SCO5286	hydrolase				GCCGTT						+						-155		

SCO4935	hypothetical protein		AGCCCG						-						-64				
SCO1652	hypothetical protein	CTCGGG						+						-267					
SCO6389	hypothetical protein		GCCCGT			GCCCGT			+			+			-282			-282	
SCO0829	serine protease		AGCCCG			AGCCCG			-			-			-115			-115	
SCO2200	hypothetical protein			TACCAA						-						-62			
SCO2201	hypothetical protein			TACCAA						-						-31			
SCO6522	hypothetical protein	GGCCGG			GGCCGG			-			-			-51			-51		
SCO5744	dihydrodipicolinate synthase	GCCGGC			GCCGGC			+			+			-200			-200		
SCO5629	ATP /GTP-binding protein		GCCCGG			GCCCGG			+			+			-194			-194	
SCO0753	hypothetical protein		GCCCGC<br>GCCCGG			GCCCGC<br>GCCCGG			+ +			+ +			-333<br>-151			-333<br>-151	
SCO4837	serine hydroxymethyltransferase		GCCCGC			GCCCGC			+			+			-211			-211	
SCO2878	hypothetical protein		CTGCCG						-						-13				
SCO6822	integral membrane efflux protein	GCCGGC<br>GGCCGG			GCCGGC<br>GGCCGG			+ -			+ -			-82 -<br>50			-82 -<br>50		
SCO5555	hypothetical protein	TGCCGC						-						-52					
SCO5000	hypothetical protein		AGCCCG			AGCCCG			-			-			-344			-344	
SCO5418	transcriptional regulator		AGCCCG						-						-231				
SCO3404	cell division protein FtsH-like protein		GCCCGT			GCCCGT			+			+			-214			-214	
SCO7740	hypothetical protein	GCCGGC			GCCGGC			+			+			-121			-121		
SCO6197	hypothetical protein		AGCCCG			AGCCCG			-			-			-119			-119	
SCO6828	hypothetical protein			CACAAG						+						-98			
SCO1301	exonuclease		GCCCGG			GCCCGG			+			+			-55			-55	
SCO6622	ATP/GTP binding protein		AGCCCG			AGCCCG			-			-			-80			-80	
SCO7747	hypothetical protein	GGCCGG			GGCCGG			-			-			-374			-374		
SCO2372	small hydrophobic protein	GGCCGG			GGCCGG			-			-			-219			-219		
SCO3895	hypothetical protein		AGCCCG			AGCCCG			-			-			-119			-119	
SCO3896	RNA nucleotidyltransferase		AGCCCG			AGCCCG			-			-			-106			-106	
SCO3413	transcriptional regulator		GCCCGG			GCCCGG			+			+			-60			-60	
SCO3564	pH-dependent sodium/proton antiporter	GGCCGG			GGCCGG			-			-			-179			-179		
SCO0895	RNA polymerase principal sigma factor HrdC		AGCCCG			AGCCCG			-			-			-31			-31	
SCO0811	ABC transporter ATP-binding protein			GACACC						+						-181			
SCO3004	hypothetical protein	GGCCGG			GGCCGG			-			-			-282			-282		
SCO2170	methyltransferase		AGCCCG			AGCCCG			-			-			-28			-28	

SCO7213	hypothetical protein		AGCCCG			AGCCCG			-			-			-293			-293	
SCO1150	50S ribosomal protein L31			CTGCTC						-						-5			
SCO1704	hypothetical protein	TGCCGG						-						-61					
SCO0588	sensor kinase		AGCCCG			AGCCCG			-			-			-102			-102	
SCO2921	hypothetical protein		AGCCCG			AGCCCG			-			-			-123			-123	
SCO5802	ATP-dependent helicase	GGCCGG						-						-111					
SCO0752	protease		GCCCGC			GCCCGC			+			+			-248			-248	
SCO4236	tRNA/rRNA methyltransferase			ACCACC						+						-51			
SCO2867	hypothetical protein		AGCCCG			AGCCCG			-			-			-57			-57	
SCO4941	hypothetical protein	GCCGGC			GCCGGC			+			+			-124			-124		
SCO7444	cytochrome P450 (fragment)	GGCCGG			GGCCGG			-			-			-13			-13		
SCO7605	metallopeptidase	GGCCGG			GGCCGG			-			-			-136			-136		
SCO1973	hypothetical protein			AACACA						+						-212			
SCO7606	amino acid binding protein	GGCCGG			GGCCGG			-			-			-63			-63		
SCO2187	hypothetical protein	GCCGGC	AGCCCG		GCCGGC	AGCCCG		+	-		+	-		-189	-208		-189	-208	
SCO0014	hypothetical protein	GGCCGG			GGCCGG			-			-			-297			-297		
SCO4034	RNA polymerase sigma factor						GCCCAC						-						-226
SCO2553	oxidoreductase		AGCCCG			AGCCCG			-			-			-41			-41	
SCO2068	alkaline phosphatase	GCCGGC			GCCGGC			+			+			-99			-99		
SCO3822	hypothetical protein	GACGGG						+						-266					
SCO4292	glucosyl-3-phosphoglycerate synthase				GTCGTG						+						-284		
SCO2062	hypothetical protein		AGCCCG			AGCCCG			-			-			-34			-34	
SCO2063	small hydrophilic protein		AGCCCG			AGCCCG			-			-			-190			-190	
SCO3713	hypothetical protein		GCCCGA			GCCCGA			+			+			-77			-77	
SCO7075	two component response regulator protein		AGCCCG			AGCCCG			-			-			-192			-192	
SCO4444	glutathione peroxidase	GCCGGC			GCCGGC			+			+			-114			-114		
SCO6614	hypothetical protein		AGCCCG						-						-75				
SCO1350	hypothetical protein	GGCCGG			GGCCGG			-			-			-213			-213		
SCO1084	thioredoxin		GCCCGT			GCCCGT			+			+			-191			-191	
SCO5310	hypothetical protein				GCCCGG						-						-267		
SCO6855	hypothetical protein	GGCCGG			GGCCGG			-			-			-80			-80		
SCO4596	two-component system response regulator	TGACGG						-						-249					
SCO6712	copper oxidase	GGCCGG			GGCCGG			-			-			-283			-283		

SCO3152	hypothetical protein	GGCCGG			GGCCGG			-			-			-115			-115		
SCO4806	hypothetical protein		AGCCCG			AGCCCG			-			-			-100			-100	
SCO3702	DNA-binding protein		GCCCGG			GCCCGG			+			+			-129			-129	
SCO7660	voltage-gated potassium channel	GGCCGG			GGCCGG			-			-			-283			-283		
SCO3159	hypothetical protein		AGCCCG			AGCCCG			-			-			-110			-110	
SCO3158	hypothetical protein		AGCCCG			AGCCCG			-			-			-75			-75	
SCO1371	oxidoreductase		GCCCGC			GCCCGC			+			+			-104			-104	
SCO3024	transporter	GCCGGC			GCCGGC			+			+			-34			-34		
SCO3010	hypothetical protein				GGACGG						-						-105		
SCO3490	transposase		GCCCGC			GCCCGC			+			+			-127			-127	
SCO1847	cobalamin biosynthesis protein		GCCCGG			GCCCGG			+			+			-190			-190	
SCO3974	hypothetical protein	GCCGGG						+						-134					
SCO5419	Thioredoxin		AGCCCG						-						-72				
SCO4818	hypothetical protein	ACCGGG						+						-176					
SCO1774	regulatory protein		GCCCGG			GCCCGG			+			+			-25			-25	
SCO0325	hypothetical protein					GCCCGG						+						-193	
SCO7348	hypothetical protein					GCCCGT						+						-45	
SCO5897	oxidase	GGCCGG			GGCCGG			-			-			-72			-72		
SCO5726	hypothetical protein		AGCCCG			AGCCCG			-			-			-58			-58	
SCO0001	hypothetical protein		AGCCCG			AGCCCG			-			-			-313			-313	
SCO5025	transcriptional regulator	GCCGGC			GCCGGC			+			+			-94			-94		
SCO3798	chromosome condensation protein		GCCCGG			GCCCGG			+			+			-75			-75	
SCO6685	two-component system response regulator				GCGCGC						-						-42		
SCO2710	polysaccharide deacetylase				GGCCGG						-						-32		
SCO0216	nitrate reductase subunit alpha NarG2		AGCCCG			AGCCCG			-			-			-399			-399	
SCO0215	hypothetical protein		AGCCCG			AGCCCG			-			-			-192			-192	
SCO4096	ATP-dependent RNA helicase	GTCGGG			GTCGGG			+			+			-250			-264		
SCO1056	sugar transport sugar binding protein			ACCCAA						-						-300			
SCO3543	DNA topoisomerase I					GCCCGC						+						-131	
SCO2716	hypothetical protein	GGCCGG			GGCCGG			-			-			-143			-143		
SCO1055	alpha-xylosidase			ACCCAA						-						-11			
SCO4157	Protease		GCCCGG			GCCCGG			+			+			-49			-49	
SCO4248	hypothetical protein		AGCCCG			AGCCCG			-			-			-39			-39	

SCO4249	hypothetical protein		AGCCCG			AGCCCG			-			-			-205			-205	
SCO2328	Dipeptidase				GAGCGT						-						-106		
SCO2255	hypothetical protein			ACCATA						+						-53			
SCO5251	acetyltransferase	GGCCGG			GGCCGG			-			-			-269			-269		
SCO6846	hypothetical protein	GGCCGG			GGCCGG			-			-			-360			-360		
SCO2256	3-methyl-2-oxobutanoate hydroxymethyltransferase			ACCATA						+						-266			
SCO1536	hypothetical protein				ACCGGG						+						-114		
SCO3145	hypothetical protein	GCCGGC			GCCGGC			+			+			-7			-7		
SCO2396	hypothetical protein		GCCCGT			GCCCGT			+			+			-95			-95	
SCO3149	dimethyladenosine transferase	GCCGGG						+						-15					
SCO3315	transcriptional regulator		GCCCGC			GCCCGC			+			+			-71			-71	
SCO1228	acyltransferase					AGCCCG						-						-168	
SCO6163	sensor kinase					ACCAGA						+						-144	
SCO3314	dehydrogenase		GCCCGC			GCCCGC			+			+			-57			-57	
SCO2093	hypothetical protein	GGCCGG			GGCCGG			-			-			-372			-372		
SCO2092	S-adenosyl-methyltransferase MraW	GGCCGG			GGCCGG			-			-			-124			-124		
SCO3026	hypothetical protein	GCCGGC			GCCGGC			+			+			-82			-82		
SCO0368	Transposase	GGCCGG			GGCCGG			-			-			-161			-161		
SCO3598	hypothetical protein				GCCGGG						+						-33		
SCO4627	hypothetical protein		GCCCGA			GCCCGA			+			+			-302			-302	
SCO0178	hypothetical protein		GCCCGT			GCCCGT			+			+			-211			-211	
SCO4789	hypothetical protein	GGCCGG			GGCCGG			-			-			-127			-127		
SCO7773	hypothetical protein	GCCGGC			GCCGGC			+			+			-174			-174		
SCO7772	hypothetical protein		AGCCCG			AGCCCG			-			-			-236			-236	
SCO4028	hypothetical protein		AGCCCG			AGCCCG			-			-			-129			-129	
SCO5157	metal-transport protein	GCCGGC			GCCGGC			+			+			-129			-129		
SCO5158	hypothetical protein	GCCGGC			GCCGGC			+			+			-394			-394		
SCO2639	RNA polymerase sigma factor		AGCCCG			AGCCCG			-			-			-98			-98	
SCO4628	Regulator		GCCCGA			GCCCGA			+			+			-124			-124	
SCO7714	acetyltransferase		AGCCCG			AGCCCG			-			-			-75			-75	
SCO4561	hypothetical protein		GCCCGG			GCCCGG			+			+			-215			-215	
SCO2950	DNA-binding protein HU (hs1)		GCCCGA			GCCCGA			+			+			-266			-266	
SCO2640	aspartate-semialdehyde dehydrogenase		AGCCCG			AGCCCG			-			-			-79			-79	

SCO4085	Lipoprotein	GCCGGG			GCCGGG			+			+			-73			-73		
SCO3925	transcriptional regulator	ACGCGG						-						-44					
SCO4027	anti sigma factor antagonist		AGCCCG			AGCCCG			-			-			-27			-27	
SCO4165	hypothetical protein		GCCCGC			GCCCGC			+			+			-30			-30	
SCO3922	hypothetical protein			TGCCAC						-						-13			
SCO3923	hypothetical protein			TGCCAC						-						-46			
SCO0196	hypothetical protein		GCCCGA			GCCCGA			+			+			-19			-19	
SCO1024	hypothetical protein		AGCCCG			AGCCCG			-			-			-42			-42	
SCO0191	lycopene cyclase		AGCCCG			AGCCCG			-			-			-23			-23	
SCO0989	hypothetical protein	GGCCGG			GGCCGG			-			-			-210			-210		
SCO1956	LacI family transcription regulator	GTCGGG						+						-206					
SCO1516	preprotein translocase subunit SecD		AGCCCG			AGCCCG			-			-			-53			-53	
SCO2006	hypothetical protein		AGCCCG			AGCCCG			-			-			-136			-136	
SCO3365	hypothetical protein	GCCGGC			GCCGGC			+			+			-237			-237		
SCO3335	AraC family transcription regulator			GGCACG						+						-63			
SCO4109	oxidoreductase				CGTCGG						-						-78		
SCO1169	xylose isomerase		GCCCGA			GCCCGA			+			+			-153			-153	
SCO1107	hypothetical protein			ATCACA						+						-89			
SCO4774	glycerol phosphate dehydrogenase		GCCCGG			GCCCGG			+			+			-253			-253	
SCO1875	penicillin binding protein					AGCCCG						-						-101	
SCO5716	peptide transport system peptide binding protein		AGCCCG			AGCCCG			-			-			-58			-58	
SCO2327	hypothetical protein				GAGCGT						-						-132		
SCO1170	xylulose kinase		GCCCGA			GCCCGA			+			+			-2			-2	
SCO6178	Deacetylase		GCCCGT			GCCCGT			+			+			-170			-170	
SCO6179	nucleotide-sugar dehydratase		GCCCGT			GCCCGT			+			+			-1			-1	
SCO0863	hypothetical protein				GCCGGC						+						-207		
SCO5287	MarR family transcriptional regulator				GCCGTT						+						-93		
SCO4780	succinic semialdehyde dehydrogenase		GCCCGC			GCCCGC			+			+			-25			-25	
SCO6606	hypothetical protein				TTCCGG						-						-41		
SCO6607	hypothetical protein				TTCCGG						-						-89		
SCO4651	Lipoprotein		GCCCGG			GCCCGG			+			+			-144			-144	
SCO2920	Protease		AGCCCG			AGCCCG			-			-			-310			-310	

SCO0138	short chain dehydrogenase		AGCCCG			AGCCCG			-			-			-40			-40	
SCO6473	crotonyl CoA reductase			ACCCAC						-						-293			
SCO2489	TetR family transcriptional regulator		AGCCCG			AGCCCG			-			-			-43			-43	
SCO7470	phenylacetic acid degradation protein PaaI	GCCGGC			GCCGGC			+			+			-83			-83		
SCO7471	phenylacetate-CoA oxygenase subunit PaaA	GCCGGC			GCCGGC			+			+			-81			-81		
SCO6609	hypothetical protein			ACCACG						+						-71			
SCO2588	hypothetical protein				CGTCGC						-						-176		
SCO0815	hypothetical protein		GCCCGG			GCCCGG			+			+			-59			-59	
SCO2427	Arabinase	GCCGGC			GCCGGC			+			+			-239			-239		
SCO6411	Hydrolase		GCCCGT			GCCCGT			+			+			-82			-82	
SCO7804	hypothetical protein				GCCGGC						+						-336		
SCO3911	replicative DNA helicase		AGCCCG			AGCCCG			-			-			-273			-273	
SCO3910	hypothetical protein		AGCCCG			AGCCCG			-			-			-177			-177	
SCO2428	phosphate binding protein	GCCGGC			GCCGGC			+			+			-102			-102		
SCO4579	hypothetical protein			CACCAC						-						-55			
SCO4960	sigma factor		GCCCGG<br>GCCCGG<br>GCCCGG			GCCCGG<br>GCCCGG<br>GCCCGG			+ + +			+ + +			-100<br>-130<br>-150			-130<br>-150<br>-100	
SCO2587	gamma-glutamyl kinase	CGCGCC<br>GCCCGG<br>GGCCGG			GGCCGG			+ - -			-			-319<br>-346<br>-137			-137		
SCO1349	hypothetical protein	GGCCGG			GGCCGG			-			-			-8			-8		
SCO6722	Regulator		AGCCCG			AGCCCG			-			-			-29			-29	
SCO5206	hydrogen peroxide sensitive repressor	GGCCGG			GGCCGG			-			-			-164			-164		
SCO1030	hypothetical protein	GGCCGG			GGCCGG			-			-			-119			-119		
SCO6781	regulatory protein	CGCCGG						-						-64					
SCO4773	nucleotide-sugar dehydrogenase		GCCCGG			GCCCGG			+			+			-49			-49	
SCO2745	LacI family transcriptional regulator				TTCGGG						+						-151		
SCO5362	hypothetical protein		CCCCGA						+						-44				
SCO2267	heme oxygenase		GGTCAG			GGTCAG			-			-			-137			-137	
SCO1375	hypothetical protein				GCGCGG						-						-290		
SCO4053	transport integral membrane protein	GGCCGG			GGCCGG			-			-			-120			-120		
SCO4475	cytochrome biogenesis-like protein	GGCCGG			GGCCGG			-			-			-137			-137		
SCO3336	Hydrolase			GGCACG						+						-11			

SCO0916	hypothetical protein	GCCGGC			GCCGGC			+			+			-62			-62		
SCO0917	Oxygenase	GCCGGC			GCCGGC			+			+			-188			-188		
SCO2232	maltose operon transcriptional repressor		GCCCGT			GCCCGT			+			+			-367			-367	
SCO2231	maltose-binding protein		GCCCGT			GCCCGT			+			+			-24			-24	
SCO6384	integral membrane lysyl-tRNA synthetase		GCCCGC			GCCCGC			+			+			-290			-290	
SCO1935	transketolase	GCCGGC						+						-78					
SCO1934	protoheme IX farnesyltransferase	GCCGGC						+						-223					
SCO5316	acyl carrier protein	GGCCGG GCCGGC			GGCCGG GCCGGC			- +			- +			-28 -27			-28 -27		
SCO0449	solute-binding lipoprotein					ACACAG						-						-196	
SCO7093	transcriptional regulator		AGCCCG			AGCCCG			-			-			-400			-400	
SCO5365	Transferase		GCCCGT			GCCCGT			+			+			-1			-1	
SCO5447	neutral zinc metalloprotease	GCCGGC			GCCGGC			+			+			-59			-59		
SCO3840	hypothetical protein	CCGCGC						-						-41					
SCO0812	sugar isomerase			GACACC						+						-95			
SCO7833	hypothetical protein	GGCCGG			GGCCGG			-			-			-295			-295		
SCO0055	membrane-associated oxidoreductase		GCCCGC			GCCCGC			+			+			-76			-76	
SCO0056	hypothetical protein		GCCCGC			GCCCGC			+			+			-9			-9	
SCO4006	long-chain-fatty-acid--CoA ligase	GGGCGG						-						-83					
SCO7310	regulatory protein	GGCCGG			GGCCGG			-			-			-174			-174		
SCO0326	hypothetical protein	GCCGGC			GCCGGC			+			+			-30			-30		
SCO2620	trigger factor	GGCCGG			GGCCGG			-			-			-277			-277		
SCO2621	hypothetical protein		GCCCGT			GCCCGT			+			+			-361			-361	
SCO2622	hypothetical protein		GCCCGT			GCCCGT			+			+			-273			-273	
SCO4068	phosphoribosylamine--glycine ligase		GCCCGC			GCCCGC			+			+			-41			-41	
SCO2188	peptidase	GCCGGC	AGCCCG		GCCGGC	AGCCCG		+	-		+	-		-235	-216		-235	-216	
SCO2077	hypothetical protein		GCCCGT			GCCCGT			+			+			-38			-38	
SCO4064	hypothetical protein		GCCCGG			GCCCGG			+			+			-170			-170	
SCO2842	hypothetical protein	ACACGA						-						-2					
SCO0324	hypothetical protein					GCCCGG						+						-89	
SCO5290	hypothetical protein		GCCCGC			GCCCGC			+			+			-39			-39	
SCO6711	hypothetical protein	GGCCGG			GGCCGG			-			-			-49			-49		
SCO2183	2-oxoacid dehydrogenase subunit E1		AGCCCG			AGCCCG			-			-			-98			-98	

SCO3512	hypothetical protein		AGCCCG			AGCCCG			-			-			-34			-34	
SCO5195	hypothetical protein		GCCCGT			GCCCGT			+			+			-136			-136	
SCO2454	hypothetical protein	GCCGGC			GCCGGC			+			+			-83			-83		
SCO5294	hypothetical protein		AGCCCG			AGCCCG			-			-			-64			-64	
SCO6257	ABC transporter sugar binding lipoprotein			ACCCCA						-						-73			
SCO4746	lipase	GCCGGC			GCCGGC			+			+			-34			-34		
SCO1009	transposase	GCCGGC			GCCGGC			+			+			-400			-400		
SCO3003	hypothetical protein	GGCCGG			GGCCGG			-			-			-250			-250		
SCO6989	hypothetical protein	GGCCGG						-						-16					
SCO2744	hypothetical protein				TTCGGG						+						-134		
SCO1085	acyltransferase		GCCCGT			GCCCGT			+			+			-74			-74	
SCO6799	L-threonine 3-dehydrogenase		AGCCCG			AGCCCG			-			-			-133			-133	
SCO5696	4-hydroxy-3-methylbut-2-en-1-yl diphosphate synthase		AGCCCG<br>GCCCGA			AGCCCG<br>GCCCGA			- +			- +			-153<br>-183			-153<br>-183	
SCO1227	DNA-binding protein					AGCCCG						-						-48	
SCO6390	hypothetical protein		GCCCGT			GCCCGT			+			+			-193			-193	
SCO7556	sugar transport permease	GCCGGC			GCCGGC			+			+			-49			-49		
SCO0638	lipoprotein	GCCGGC			GCCGGC			+			+			-115			-115		
SCO6153	regulatory protein	GGCCGT		GCCCCG				-		+				-397		-234			
SCO0375	integral membrane transport protein		GCCCGC			GCCCGC			+			+			-86			-86	
SCO7746	insertion element transposase	GGCCGG			GGCCGG			-			-			-267			-267		
SCO4176	hypothetical protein		GCCCGG			GCCCGG			+			+			-67			-67	
SCO0322	ABC transport ATP-binding subunit	GCCGTC						+						-99					
SCO5168	hypothetical protein	GCCGGC			GCCGGC			+			+			-23			-23		
SCO4175	hypothetical protein		GCCCGG			GCCCGG			+			+			-104			-104	
SCO4173	hypothetical protein		CGCCCG						-						-37				
SCO3856	peptidyl-prolyl cis-trans isomerase			ACCACT						+						-184			
SCO3857	regulatory protein			ACCACT						+						-136			
SCO7784	oxidoreductase		GCCCGC			GCCCGC			+			+			-24			-24	
SCO5163	hypothetical protein		AGCCCG			AGCCCG			-			-			-71			-71	
SCO7225	chitinase			TCCCCC						-						-209			
SCO7224	hypothetical protein			TCCCCC						-						-101			
SCO1421	hypothetical protein		GCCCGG			GCCCGG			+			+			-15			-15	

SCO5164	hypothetical protein		AGCCCG			AGCCCG			-			-			-164			-164	
SCO7659	oxidoreductase	GGCCGG			GGCCGG			-			-			-43			-43		

Table S2 Primers sequence

Gene	Forward	Reverse
SCO6685	GAACGCTTCCTGGACGAGA	CATGTAGTTGCGGACCGTC
SCO2571	TCAACTCCTGGTACGACGAC	CTTGAAGACGGGGAAGTTGC
SCO6164	CGCGTCACCCAGCTTCAG	CGCGTGTAGGGGAGGATC
SCO5820	CGTCGAGGGTGTTCGGCTG	CGCGAGCCCATCTCGCTGC
SCO3732	CTCAAGGACCTCATCGACCG	TCCAGGAACATGATGACCCG
SCO2077	GTTCCGAGGCCAACAAGATC	ACTCCAGGTACGACTTCAGC
SCO2964	ACAAGACACGCAATCCCATC	CACTTGTGGACGGACATCAC
SCO3911	CTCTACATCGACGACTCCCC	CGCGGTGACTCCTTCTCATA
SCO2716	CTCGTCCTCGTCCTCGACT	GCGTCGTTCTCGCACTTG
SCO2950	AGGTTGTCGGCGACATCGT	CGGAGACCTTGACGCTGTA
SCO5881	AAACGTCGGTCGAAGAACTG	GTCTTGCCCTGGGTCAGTAA
SCO5085	TAATTTCGCATCCGCTGAAC	CTACACGAGCACCTTCTCACC
16S	TGCCAGCAGCCGCGGTAATA	GACTGCAGACCCGGGGTTAA
